# Causality of immune cells on primary sclerosing cholangitis: a bidirectional two-sample Mendelian randomization study

**DOI:** 10.3389/fimmu.2024.1395513

**Published:** 2024-07-01

**Authors:** Pu Wu, Sinan Xie, Yunshi Cai, Hu Liu, Yinghao Lv, Ying Yang, Yucheng He, Bangjie Yin, Tian Lan, Hong Wu

**Affiliations:** ^1^ Department of General Surgery, West China Hospital, Sichuan University, Chengdu, China; ^2^ Liver Transplant Center, Transplant Center, West China Hospital, Sichuan University, Chengdu, China; ^3^ Laboratory of Hepatic AI Translation, Frontiers Science Center for Disease-Related Molecular Network, West China Hospital, Sichuan University, Chengdu, China

**Keywords:** primary sclerosing cholangitis (PSC), Mendelian randomization (MR), immune cells, genome-wide association study (GWAS), causal effect

## Abstract

**Background:**

Observational studies have indicated that immune dysregulation in primary sclerosing cholangitis (PSC) primarily involves intestinal-derived immune cells. However, the causal relationship between peripheral blood immune cells and PSC remains insufficiently understood.

**Methods:**

A bidirectional two-sample Mendelian randomization (MR) analysis was implemented to determine the causal effect between PBC and 731 immune cells. All datasets were extracted from a publicly available genetic database. The standard inverse variance weighted (IVW) method was selected as the main method for the causality analysis. Cochran’s *Q* statistics and MR-Egger intercept were performed to evaluate heterogeneity and pleiotropy.

**Results:**

In forward MR analysis, the expression ratios of CD11c on CD62L+ myeloid DC (OR = 1.136, 95% CI = 1.032–1.250, *p* = 0.009) and CD62L-myeloid DC AC (OR = 1.267, 95% CI = 1.086–1.477, *p* = 0.003) were correlated with a higher risk of PSC. Each one standard deviation increase of CD28 on resting regulatory T cells (Treg) (OR = 0.724, 95% CI = 0.630–0.833, *p* < 0.001) and CD3 on secreting Treg (OR = 0.893, 95% CI = 0.823–0.969, *p* = 0.007) negatively associated with the risk of PSC. In reverse MR analysis, PSC was identified with a genetic causal effect on EM CD8+ T cell AC, CD8+ T cell AC, CD28− CD127− CD25++ CD8+ T cell AC, CD28− CD25++ CD8+ T cell AC, CD28− CD8+ T cell/CD8+ T cell, CD28− CD8+ T cell AC, and CD45 RA− CD28− CD8+ T cell AC.

**Conclusion:**

Our study indicated the evidence of causal effects between PSC and immune cells, which may provide a potential foundation for future diagnosis and treatment of PSC.

## 1 Introduction

Primary sclerosing cholangitis (PSC) is an uncommon cholestatic liver disorder with an unknown cause. Recent evidence suggests that its prevalence in Northern Europe and North America varies between 3.85 and 16.2 cases per 100,000 individuals, showing an upward trend over time (1). Pathological manifestation is characterized by onion skin fibrosis encircling the bile ducts and typically presenting as beaded stenosis along with dilation of intrahepatic or/and extrahepatic bile ducts on imaging studies. The natural progression of PSC exhibits considerable heterogeneity and unpredictability; nevertheless, a significant portion of patients ultimately advance towards end-stage liver disease requiring exclusive treatment through liver transplantation (LT) (2–5).

The pathogenesis of PSC remains incompletely understood and likely results from the intricate interplay between genetic and environmental factors. Recent comprehensive genome-wide association studies (GWASs) have successfully identified a total of 23 genetic risk loci, encompassing both HLA and non-HLA genes, such as Fut2 gene, which are significantly associated with PSC development (6, 7). These findings provide compelling evidence for the substantial contribution of genetic factors to the pathogenesis of PSC. As an autoimmune liver disease, previous research has demonstrated robust HLA associations in genetic studies, suggesting the involvement of adaptive immune response mechanisms (4). Intriguingly, extensive fine-mapping efforts coupled with functional annotations have revealed numerous novel loci primarily linked to immune function, particularly T helper cell subsets including Th17, Th1, and Th2 cells. However, the causal relationship between specific immune cell types and the initiation of PSC still remains elusive.

Mendelian randomization (MR) analysis is a robust methodology that employs genetic variation as an instrumental variable (IV) to establish causal relationships between risk factors and diseases (8, 9). By adhering to Mendel’s law of independent assortment, genetic variants are typically assumed to be independent of each other and unaffected by confounding variables. Consequently, the correlation ratio in MR studies was assessed against randomized clinical trial (RCT) findings for enhanced reliability (10). In this study, we used a two-sample MR analysis to detect the potential causal association between immune cells and PSC.

## 2 Materials and methods

### 2.1 Study design

**Figure 1:**
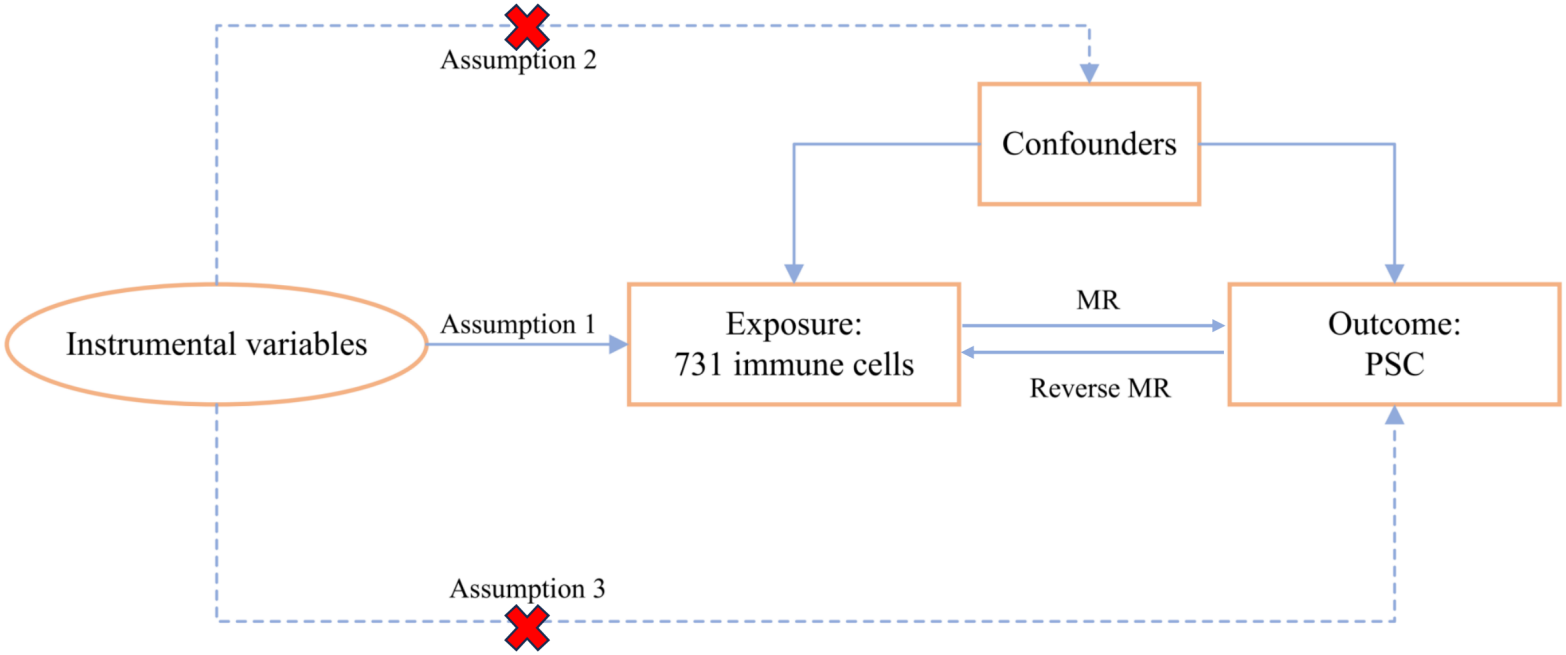
The study design of the MR analysis.

We systematically evaluated the causal association between 731 immune cell signatures and PSC using a bidirectional two-sample MR analysis. In an MR study, the IVs must adhere to three fundamental assumptions for the validity of causal inference: (1) Relevance assumption: IVs were significantly associated with exposure, (2) Independence assumption: IVs were independent of potential confounders, and (3) Exclusivity assumption: IVs could affect the PSC solely through immune cells. The overall MR design is depicted in **Figure 1**.

### 2.2 Data sources

PSC cases were extracted from a publicly available GWAS database (https://gwas.mrcieu.ac.uk/) from the DIAGRAM Consortium including 14,890 individuals (2,871 cases and 12,019 controls) of European ancestry. All cases included in this cohort conformed to the American Association for the Study of Liver Diseases criteria for PSC. The full GWAS summary statistics for each immunophenotype with accession numbers from GCST90001391 to GCST90002121 are publicly available from the GWAS Catalog (11). A total of 731 immunophenotypes included absolute counts (ACs) (*n* = 118), median fluorescence intensities (MFIs) (*n* = 389), morphological parameters (*n* = 32), and relative counts (RCs) (*n* = 192). Specifically, in the current study, we obtained B cells, classical dendritic cells (CDCs), maturation stages of T cells, monocytes, myeloid cells, TBNK (T cells, B cells, natural killer cells), and regulatory T cell (Treg) panels (12).

### 2.3 Instrumental variable selection

The significance level of genetic IVs for each immune trait was set to a genome-wide association significance level of *p*-value < 5.00E−8 (13, 14). To obtain independent variants, the extracted IVs were clumped based on the 1000 Genomes Project linkage disequilibrium (LD) structure with a threshold of *R* ^2^ < 0.001 in a 10,000-kb distance. We extracted these IVs from the outcome dataset and excluded palindromic single-nucleotide polymorphisms (SNPs) with moderate minor allele frequency (MAF) with a threshold set to 0.01. When certain exposure-related SNPs were not available in the outcome dataset, we replaced them with a suitable proxy SNP that was highly correlated with the exposure based on European ancestry (*R* ^2^ > 0.8). Additionally, the *F*-statistic was calculated for each SNP to avoid the bias of weal tool (*F* < 10) and reserve the strong instruments for the following analysis.

### 2.4 Two-sample Mendelian randomization

In our study, the inverse variance weighted (IVW), MR-Egger regression, weighted median, weighted mode, and simple mode methods were performed to infer the causal relationship, whereas the IVW method was selected as the main approach to estimate the causal effect between 731 immunophenotypes and PSC (15). The heterogeneity of the IVs was evaluated via Cochran’s *Q* test (*p* < 0.05) using the IVW method. The presence of pleiotropy was detected using the MR-Egger intercept in the MR-Egger regression method (*p* < 0.05) if the intercept significantly deviated from the origin. The leave-one-out sensitivity test using the IVW method was used to examine whether a single SNP caused the association. Additionally, scatter plots were performed to determine effect estimates.

All statistical analyses were performed using the “TwoSampleMR” package in R software (version 4.0.3).

## 3 Results

### 3.1 Exploration of the causal effect of immune traits on PSC using forward Mendelian randomization

**Figure 2:**
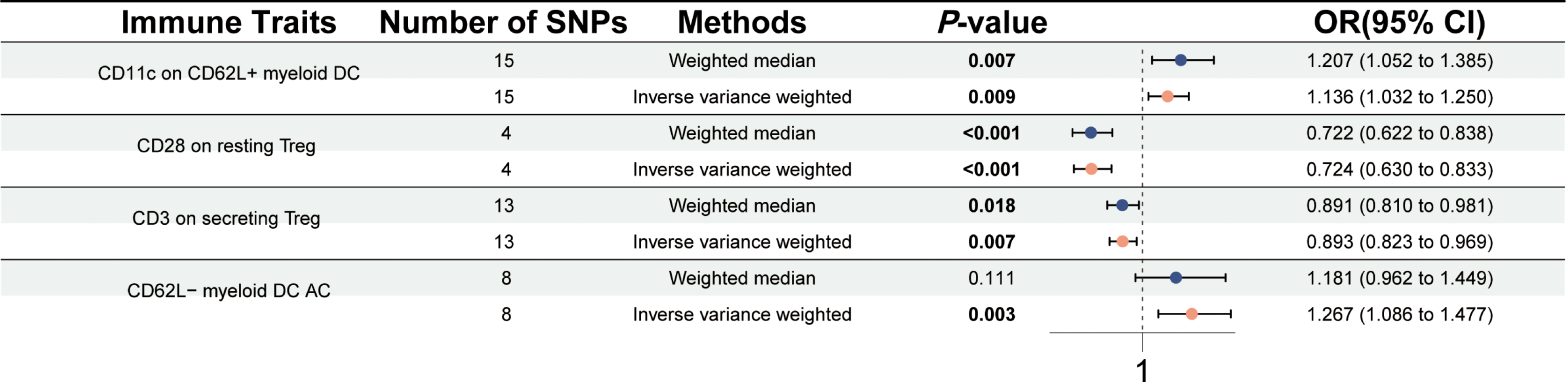
Forest plot for the causal effects of immune cells on PSC by Mendelian randomization analysis.

A two-sample MR study was performed to explore the association between 731 immune traits and PSC. A significant causal association was observed between four immune traits [CD3 on secreting Treg, CD11c on CD62L+ myeloid dendritic cell (DC), CD28 on resting Treg, and CD62L− myeloid DC AC] and PSC at a significance of 0.01 using the IVW method (**Figure 2**). The level of CD11c on CD62L+ myeloid DC was positively correlated with the risk of PSC using the IVW method (OR = 1.136, 95% CI = 1.032–1.250, *p* = 0.009) and the weighted median method (OR = 1.207, 95% CI = 1.052–1.385, *p* = 0.007). The levels of CD28 on resting Treg (OR = 0.724, 95% CI = 0.630–0.833, *p* < 0.001) and CD3 on secreting Treg (OR = 0.893, 95% CI = 0.823–0.969, *p* = 0.007) negatively associated with the risk of PSC in the IVW method. Significant and similar results were also observed in the weighted median method (CD28 on resting Treg, OR = 0.722, 95% CI = 0.622–0.838, *p* < 0.001; CD3 on secreting Treg, OR = 0.891, 95% CI = 0.810–0.981, *p* = 0.018). The higher level of CD62L− myeloid DC AC may predict a higher risk of PSC in both the IVW method (OR = 1.267, 95% CI = 1.086–1.477, *p* = 0.003) and the weighted median method (OR = 1.181, 95% CI = 0.962–1.449, *p* = 0.111). There was no significant horizontal pleiotropy in the MR-Egger intercept test (*p* > 0.05) (**Supplementary Table 1**). No heterogeneity was identified using Cochran’s *Q* test (*Q p*-value > 0.05) (**Supplementary Table 1**). Scatter plots and leave-one-out sensitivity analysis also indicated the stability of the above results (**Supplementary Figures 1** and **2**).

### 3.2 Exploration of the causal effect of PSC on immune traits using reverse Mendelian randomization

**Figure 3:**
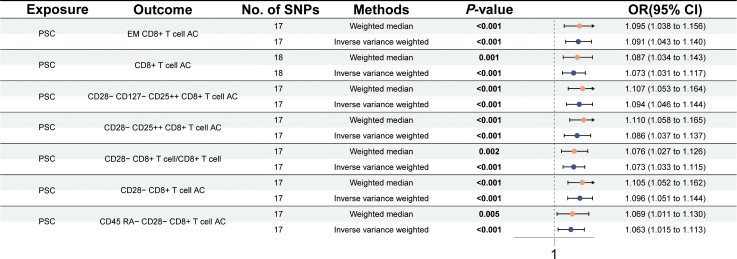
Forest plot for the causal effects of PSC on immune cells by Mendelian randomization analysis.

Subsequently, we further carried out a reverse MR study to test the causal effects of PSC on immune traits. The reverse MR study revealed that PSC onset could increase the levels of effector memory (EM) CD8+ T cell AC (OR = 1.091, 95% CI = 1.043–1.140, *p* < 0.001), CD8+ T cell AC (OR = 1.073, 95% CI = 1.031–1.117, *p* = 0.001), CD28− CD127− CD25++ CD8+ T cell AC (OR = 1.094, 95% CI = 1.046–1.144, *p* < 0.001), CD28− CD25++ CD8+ T cell AC (OR = 1.086, 95% CI = 1.037–1.137, *p* < 0.001), CD28− CD8+ T cell/CD8+ T cell (OR = 1.073, 95% CI = 1.033–1.115, *p* < 0.001), CD28− CD8+ T cell AC (OR = 1.096, 95% CI = 1.051–1.144, *p* < 0.001), and CD45 RA− CD28− CD8+ T cell AC (OR = 1.063, 95% CI = 1.015–1.113, *p* < 0.001) using the IVW method at a significance of 0.001 (**Figure 3**). Similar trends with weighted median methods were observed (EM CD8+ T cell AC, OR = 1.095, 95% CI = 1.038–1.156, *p* = 0.001; CD8+ T cell AC, OR = 1.087, 95% CI = 1.034–1.143, *p* = 0.001; CD28− CD127− CD25++ CD8+ T cell AC, OR = 1.107, 95% CI = 1.053–1.164, *p* < 0.001; CD28− CD25++ CD8+ T cell AC, OR = 1.110, 95% CI = 1.058–1.165, *p* < 0.001; CD28− CD8+ T cell/CD8+ T cell, OR = 1.076, 95% CI = 1.027–1.126, *p* = 0.002; CD28− CD8+ T cell AC, OR = 1.105, 95% CI = 1.052–1.162, *p* < 0.001; CD45 RA− CD28− CD8+ T cell AC, OR = 1.069, 95% CI = 1.011–1.130, *p* = 0.005) (**Supplementary Table 2**). Scatter plots and leave-one-out sensitivity analysis are presented in **Supplementary Figures 3** and **4**. No pleiotropy and heterogeneity were detected in the MR-Egger intercept test and Cochran’s *Q* test (*p* > 0.05) (**Supplementary Table 2**).

## 4 Discussion

Even though multiple factors were thought to be involved in PSC, immune system dysregulation played a pivotal role. Existing evidence suggests the involvement of T cells in disease development, as indicated by robust HLA associations and imbalances between Treg and Th17 cells (16–18). In our study, employing two-way MR analysis, we investigated the causal relationship between immune cell populations and the onset of PSC. Within the context of PSC background, we identified a causative association between seven distinct types of immune cells and PSC initiation; notably, various subsets of CD8+ T cells were specifically linked to the initiation of PSC.

In our study, we found that a positive correlation between CD8+ T cells and the onset of PSC when considering PSC as an exposure factor, which was consistent with previous experimental data in mice. Daniel et al. demonstrated that activated CD8+ T cells in gut-associated lymphoid tissue (GALT) induce immune-mediated cholangitis in an antigen-dependent manner. Furthermore, this study provided novel evidence for an association between colitis and cholangitis in an antigen-dependent mouse model (19). Moreover, previous studies have also demonstrated an upregulation of interferon (IFN)-γ responses in patients with PSC and mouse models of sclerosing cholangitis. IFN-γ has been shown to modulate the phenotype of hepatic CD8+ T lymphocytes and NK cells, enhancing their cytotoxicity. Conversely, attenuation of IFN-γ signaling reduced hepatocellular apoptosis, diminished the frequency of inflammatory macrophages in the liver, and ameliorated liver fibrosis. In murine models of PSC, stimulation of Treg amplification through the use of interleukin (IL)-2/anti-IL-2 immune complexes resulted in reduced CD8+ T-cell counts and improved biliary tract damage as well as fibrosis in Mdr2−/− mice (20). Therefore, targeting IFN-γ-dependent immune responses might serve as a potential therapeutic strategy for managing sclerosing cholangitis (21).

The previous studies conducted on immune cells in patients with PSC have demonstrated that the non-coding PSC risk variants demonstrate a significant enrichment in immune-specific enhancers, particularly those associated with the T-cell response to antigen stimulation. In total, they observed differential activities in 250 genes and 10,000 regulatory elements between patients and control (22). Moreover, through single-cell sequencing of T cells in the liver of patients with PSC, Tobias et al. observed a preferential inclination of naive CD4+ T cells in the hepatic microenvironment towards Th17 polarization rather than Foxp3+ regulatory T-cell differentiation, potentially implicating their involvement in PSC pathogenesis and serving as potential targets for novel therapeutic interventions (23). However, this distinctive cellular subset was not detected within our experimental findings.

Similarly, macrophages play a pivotal role in the pathogenesis of PSC. Notably, Maria et al. found an augmentation in peribiliary proinflammatory (M1-like) macrophages as well as selectively activated (M2-like) monocyte-derived macrophages within PSC compared to normal liver tissue (24). Importantly, inhibition of monocyte-derived macrophage recruitment through genetic or pharmacological depletion of CCR2 effectively prevented biliary tract injury and fibrosis. In Tiffany’s study (25), their results suggested that FXR controlled the macrophage–Th1/17 axis crucial for SC progression. Liver macrophages served as cellular targets for systemic FXR agonists in cholestatic liver disease.

The pathogenesis of PSC may also involve the participation of inflammatory factors derived from immune cells. The production of IL-1β and IL-6 by monocytes, which was crucial for Th17 differentiation, was found to be significantly elevated in patients with PSC compared to healthy controls. Furthermore, the levels of IL-1β were notably increased in patients with PSC with *Candida albicans*-stimulated peripheral blood mononuclear cells (PBMCs), surpassing both healthy controls and patients with PSC (26). Additionally, a bioinformatics-based and clinical hepatic puncture immunohistochemical study revealed a positive correlation between ANXA1 expression and the presence of chemokines, chemokine receptors, and immune cell infiltration in the liver of patients diagnosed with PSC (27).

In addition to the promotional effect of immune cells in the liver and peripheral blood on the progression of PSC, emerging evidence suggested that immune cells originating from gut might also contribute to PSC pathogenesis. Investigations have revealed that gut and liver memory T cells of common clonal origin are present in patients with PSC-IBD (28). It was also suggested that the migration of T cells from the intestine to the liver contributes to PSC pathogenesis, as previously suggested by aberrant expression of gut-specific molecules such as endothelial adhesion molecule MAdCAM-1 and chemokine CCL25, alongside α4β7+CCR9+ effector memory T cells in the inflamed liver of patients with PSC (29, 30).

Despite the MR design being less prone to confounding factors compared to other observational studies, our study has certain limitations. Although there was a strong association between immune cells and PSC (11), our study results only considered a European population and could not be immediately generalized to other ethnic groups and populations. Secondly, the genome-wide aggregated database of association studies used in this study, however, did not provide individual-level data, thereby constraining the ability to conduct subgroup analyses based on variables such as age, sex, duration of disease, treatment, and type of disease. Thirdly, the incidence of PSC may exhibit variations across different ethnicities; however, the SNP data utilized in our study were exclusively obtained from the Gaocasoid race. Consequently, it is imperative to bolster our conclusion with additional SNP data specific to other racial groups.

Overall, this exploratory study provided the latest insight into the causal association between immune cells and PSC and opened up new paths for researchers to investigate the biological mechanisms of PSC. The significance of immune cells in PSC was of great significance for future research and clinical practice. Further research on the role of phenotypic characteristics of immune cells could be conducive to the screening and guide to exploration of earlier intervention of PSC. Therefore, our objective was to identify specific immunophenotypes that may impact PSC episodes and explore prognostic and predictive biomarkers to achieve more precise treatment for patients with clinical PSC.

## 5 Conclusion

Our findings demonstrated a significant and robust causal relationship between immune cells and PSC, underscoring the imperative of incorporating the regulation of immune cell surveillance in clinical management strategies for PSC.

## Data availability statement

The datasets presented in this study can be found in online repositories. The names of the repository/repositories and accession number(s) can be found in the article/**Supplementary Material**.

## Author contributions

PW: Writing – original draft. SX: Writing – original draft. TL: Writing – review & editing. YC: Writing – review & editing. HL: Writing – review & editing. YL: Writing – review & editing. YY: Writing – review & editing. YH: Writing – review & editing. BY: Writing – review & editing. HW: Writing – review & editing.

## Supplementary material

Supplementary Figure 1 Scatter plots of MR analysis for the causal effects of immune cells on PSC. **(A)** CD11c on CD62L+ myeloid DC on PSC; **(B)** CD28 on resting Treg on PSC; **(C)** CD3 on secreting Treg on PSC; **(D)** CD62L− myeloid DC AC on PSC.

Supplementary Figure 2 The leave-one-out analyses of MR analysis for the causal effects of immune cells on PSC. **(A)** CD11c on CD62L+ myeloid DC on PSC; **(B)** CD28 on resting Treg on PSC; **(C)** CD3 on secreting Treg on PSC; **(D)** CD62L− myeloid DC AC on PSC.

Supplementary Figure 3 Scatter plots of MR analysis for the causal effects of PSC on immune cells. **(A)** PSC on EM CD8+ T cell AC; **(B)** PSC on CD8+ T cell AC; **(C)** PSC on CD28− CD127− CD25++ CD8+ T cell AC; **(D)** PSC on CD28− CD25++ CD8+ T cell AC; **(E)** PSC on CD28− CD8+ T cell/CD8+ T cell; **(F)** PSC on CD28− CD8+ T cell AC; **(G)** PSC on CD45 RA− CD28− CD8+ T cell AC.

Supplementary Figure 4 The leave-one-out analyses of MR analysis for the causal effects of PSC on immune cells. **(A)** PSC on EM CD8+ T cell AC; **(B)** PSC on CD8+ T cell AC; **(C)** PSC on CD28− CD127− CD25++ CD8+ T cell AC; **(D)** PSC on CD28− CD25++ CD8+ T cell AC; **(E)** PSC on CD28− CD8+ T cell/CD8+ T cell; **(F)** PSC on CD28− CD8+ T cell AC; **(G)** PSC on CD45 RA− CD28− CD8+ T cell AC.

Supplementary Table 1 The causal effects of immune cells on PSC by Mendelian randomization analysis.

Supplementary Table 2 The causal effects of PSC on immune cells by Mendelian randomization analysis.
