# Supplementary figures and images for: Causality of immune cells on primary sclerosing cholangitis: a bidirectional two-sample Mendelian randomization study
Source: Front Immunol. 2024 Jul 1;15:1395513. doi: 10.3389/fimmu.2024.1395513 (PMC11246896; doi:10.3389/fimmu.2024.1395513)

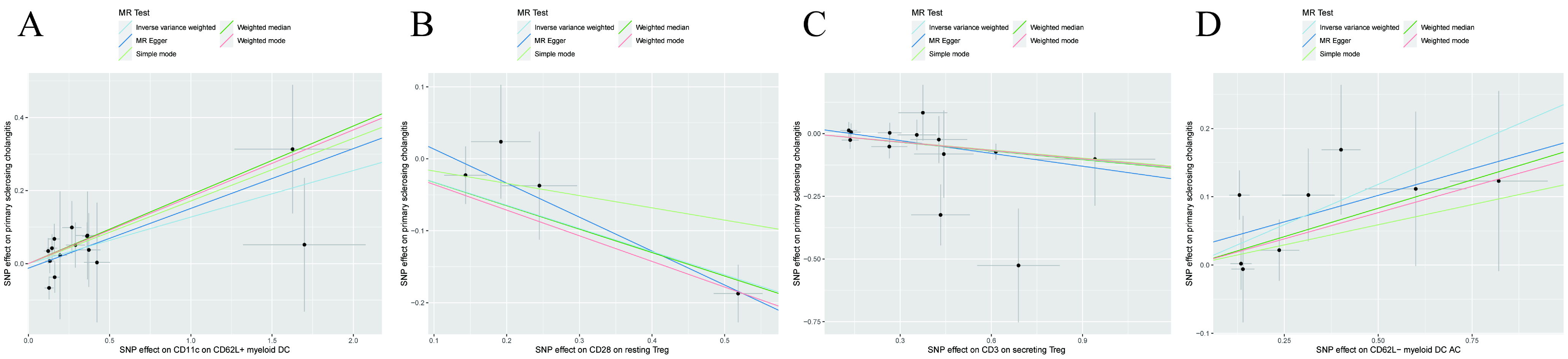

Supplement: Supplementary Figure 1 — Scatter plots of MR analysis for the causal effects of immune cells on PSC. (A) CD11c on CD62L+ myeloid DC on PSC; (B) CD28 on resting Treg on PSC; (C) CD3 on secreting Treg on PSC; (D) CD62L− myeloid DC AC on PSC. [file Image_1.tif]

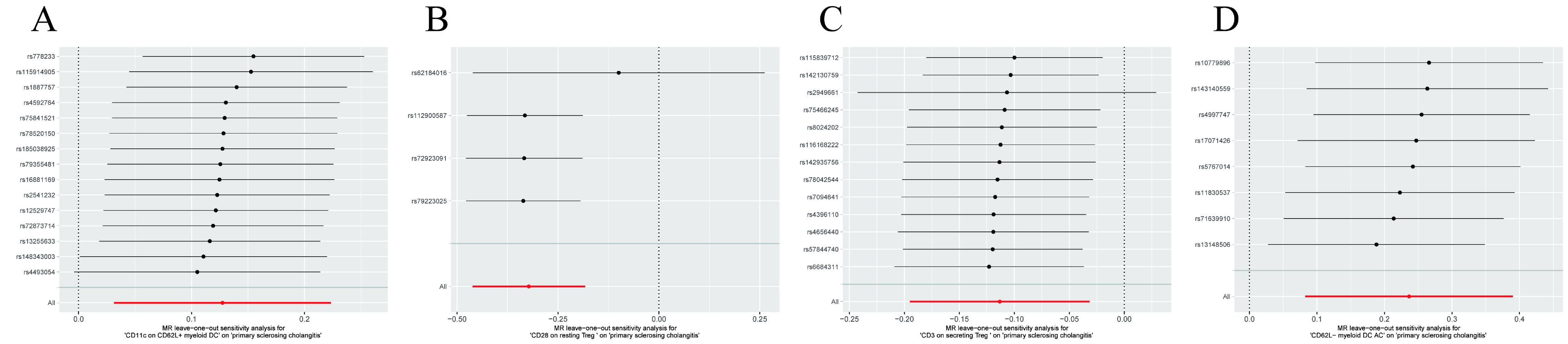

Supplement: Supplementary Figure 2 — The leave-one-out analyses of MR analysis for the causal effects of immune cells on PSC. (A) CD11c on CD62L+ myeloid DC on PSC; (B) CD28 on resting Treg on PSC; (C) CD3 on secreting Treg on PSC; (D) CD62L− myeloid DC AC on PSC. [file Image_2.tif]

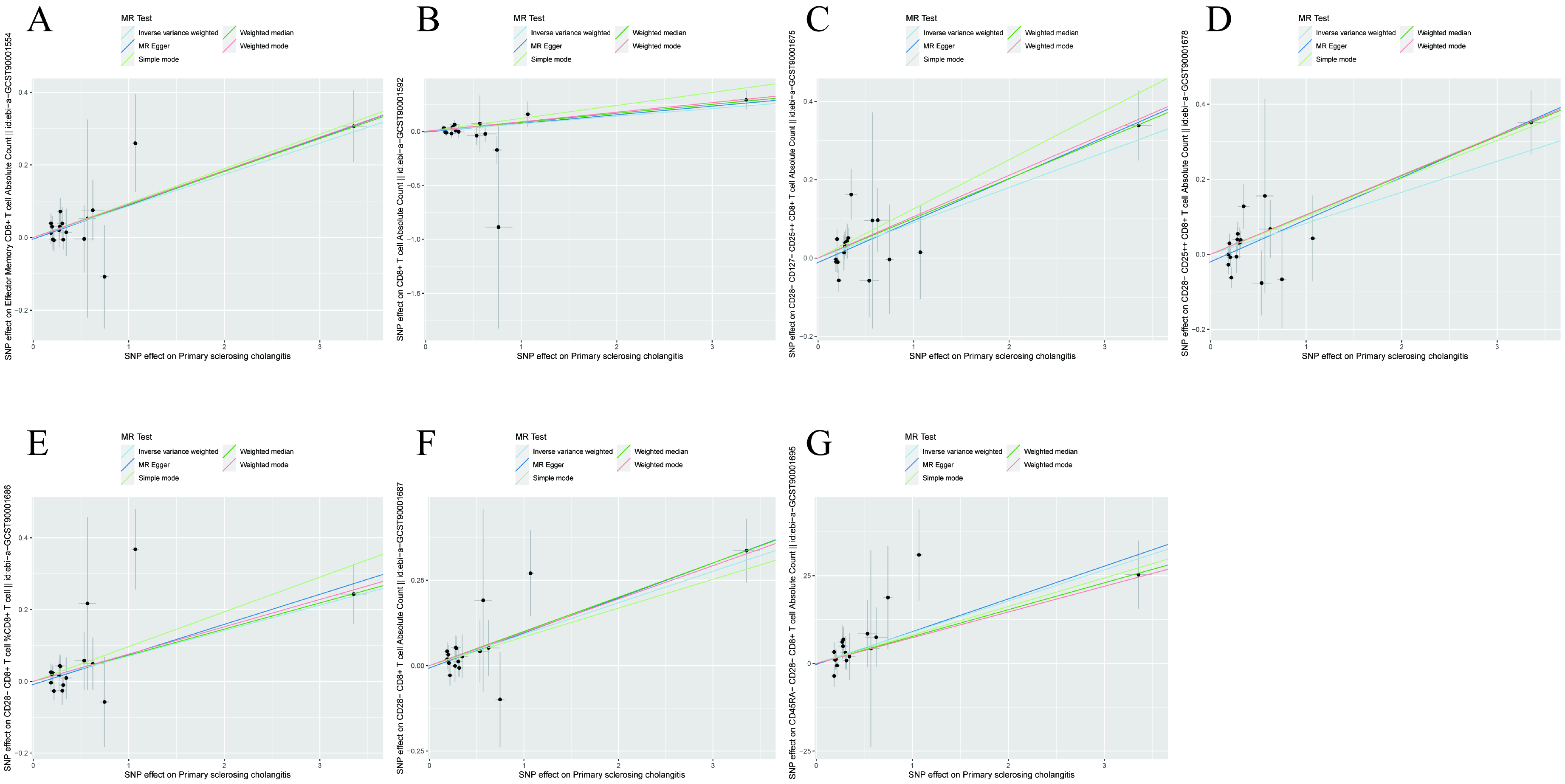

Supplement: Supplementary Figure 3 — Scatter plots of MR analysis for the causal effects of PSC on immune cells. (A) PSC on EM CD8+ T cell AC; (B) PSC on CD8+ T cell AC; (C) PSC on CD28− CD127− CD25++ CD8+ T cell AC; (D) PSC on CD28− CD25++ CD8+ T cell AC; (E) PSC on CD28− CD8+ T cell/CD8+ T cell; (F) PSC on CD28− CD8+ T cell AC; (G) PSC on CD45 RA− CD28− CD8+ T cell AC. [file Image_3.tif]

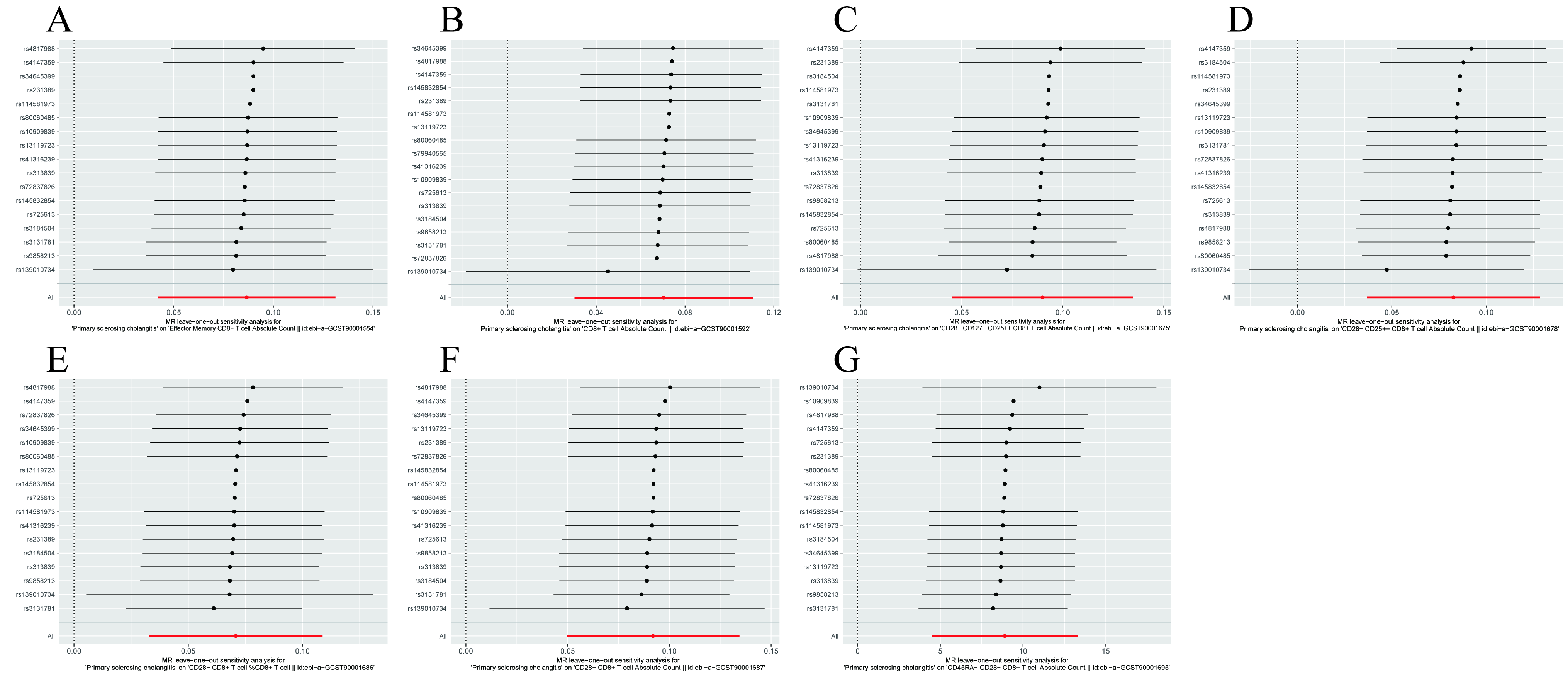

Supplement: Supplementary Figure 4 — The leave-one-out analyses of MR analysis for the causal effects of PSC on immune cells. (A) PSC on EM CD8+ T cell AC; (B) PSC on CD8+ T cell AC; (C) PSC on CD28− CD127− CD25++ CD8+ T cell AC; (D) PSC on CD28− CD25++ CD8+ T cell AC; (E) PSC on CD28− CD8+ T cell/CD8+ T cell; (F) PSC on CD28− CD8+ T cell AC; (G) PSC on CD45 RA− CD28− CD8+ T cell AC. [file Image_4.tif]
